# Tp47-Induced Monocyte-Derived Microvesicles Promote the Adherence of THP-1 Cells to Human Umbilical Vein Endothelial Cells via an ERK1/2–NF-κB Signaling Cascade

**DOI:** 10.1128/spectrum.01888-23

**Published:** 2023-06-29

**Authors:** M. Wang, J.-W. Xie, Y.-W. Zheng, X.-T. Wang, D.-Y. Yi, Y. Lin, M.-L. Tong, L.-R. Lin

**Affiliations:** a Center of Clinical Laboratory, Zhongshan Hospital of Xiamen University, School of Medicine, Xiamen University, Xiamen, China; b Institute of Infectious Disease, School of Medicine, Xiamen University, Xiamen, China; c Department of Basic Medical Science, Xiamen Medical College, Xiamen, China; Southern Medical University

**Keywords:** *Treponema pallidum*, human umbilical vein endothelial cell, THP-1 cell, microvesicles, intercellular adhesion molecule 1, vascular cell adhesion molecule 1

## Abstract

The Treponema pallidum membrane protein Tp47 induces immunocyte adherence to vascular cells and contributes to vascular inflammation. However, it is unclear whether microvesicles are functional inflammatory mediators between vascular cells and immunocytes. Microvesicles that were isolated from Tp47-treated THP-1 cells using differential centrifugation were subjected to adherence assays to determine the adhesion-promoting effect on human umbilical vein endothelial cells (HUVECs). Intercellular adhesion molecule 1 (ICAM-1) and vascular cell adhesion molecule 1 (VCAM-1) levels in Tp47-induced microvesicle (Tp47-microvesicle)-treated HUVECs were measured, and the related intracellular signaling pathways of Tp47-microvesicle-induced monocyte adhesion were investigated. Tp47-microvesicles promoted THP-1 cell adhesion to HUVECs (*P < *0.01) and upregulated ICAM-1 and VCAM-1 expression in HUVECs (*P < *0.001). The adhesion of THP-1 cells to HUVECs was inhibited by anti-ICAM-1 and anti-VCAM-1 neutralizing antibodies. Tp47-microvesicle treatment of HUVECs activated the extracellular signal-regulated kinase 1/2 (ERK1/2) and NF-κB signaling pathways, whereas ERK1/2 and NF-κB inhibition suppressed the expression of ICAM-1 and VCAM-1 and significantly decreased the adhesion of THP-1 cells to HUVECs.

**IMPORTANCE** Tp47-microvesicles promote the adhesion of THP-1 cells to HUVECs through the upregulation of ICAM-1 and VCAM-1 expression, which is mediated by the activation of the ERK1/2 and NF-κB pathways. These findings provide insight into the pathophysiology of syphilitic vascular inflammation.

## INTRODUCTION

Syphilis is a chronic, complex, sexually transmitted disease caused by the spirochetal bacterium Treponema pallidum in humans (1) and is characterized by pathological features of vascular association, including periarteritis and endarteritis, that present with significant predominantly monocytic and neutrophilic perivascular inflammatory infiltrates (2, 3). Recognition of the mechanisms by which T. pallidum promotes the adherence of monocytes to human umbilical vein endothelial cells (HUVECs) would provide important insights into syphilitic vascular inflammation. The key vascular pathology in syphilis is evinced by the T. pallidum-induced activation of human vascular endothelial cells for the upregulation of leukocyte adhesion molecule expression (4–6). Although T. pallidum lacks lipopolysaccharides, it contains abundant proinflammatory lipoproteins. The most prevalent T. pallidum protein, the Treponema pallidum membrane protein Tp47, has no sequence similarity to any other bacterial or eukaryotic protein (7). Despite several reports that Tp47 significantly causes the inflammatory reaction induced by T. pallidum (8–10), the pathogenic mechanisms of cell adhesion and intercellular signaling of Tp47 in vascular inflammation remain unclear.

Extracellular vesicles constitute a heterogeneous set of cell-derived membranous structures, including exosomes and microvesicles, that are intercellular biomessengers that enable the cell-cell interchange of proteins, lipids, and genetic material (11). Elucidation of the pathophysiological functions and medical applications involving the use or analysis of the above-mentioned vesicles is essential for an understanding of the cellular mechanisms that govern extracellular vesicle biology (12, 13). Extracellular vesicles released from endothelial cells or monocytes generate targeted cross talk between endothelial cells and monocytes (14, 15). Our previous investigations have demonstrated that both T. pallidum and Tp47 induce the adherence of a human monocytic cell line (THP-1) to human dermal vascular smooth muscle cells by increasing the levels of adhesion-associated cytokines (2, 10). It is intriguing whether microvesicles constitute a functional inflammatory mediator of the vascular cell-immunocyte interaction in vascular inflammation.

Here, a series of experiments was conducted to investigate the effect of monocyte-derived Tp47-induced microvesicles (Tp47-microvesicles) on THP-1 cell adherence to HUVECs. Furthermore, the levels of intercellular adhesion molecule 1 (ICAM-1) and vascular cell adhesion molecule 1 (VCAM-1) expression and the signaling pathways involved in cell adhesion were analyzed.

## RESULTS

### Identification of microvesicles derived from THP-1 cells.

**FIG 1:**
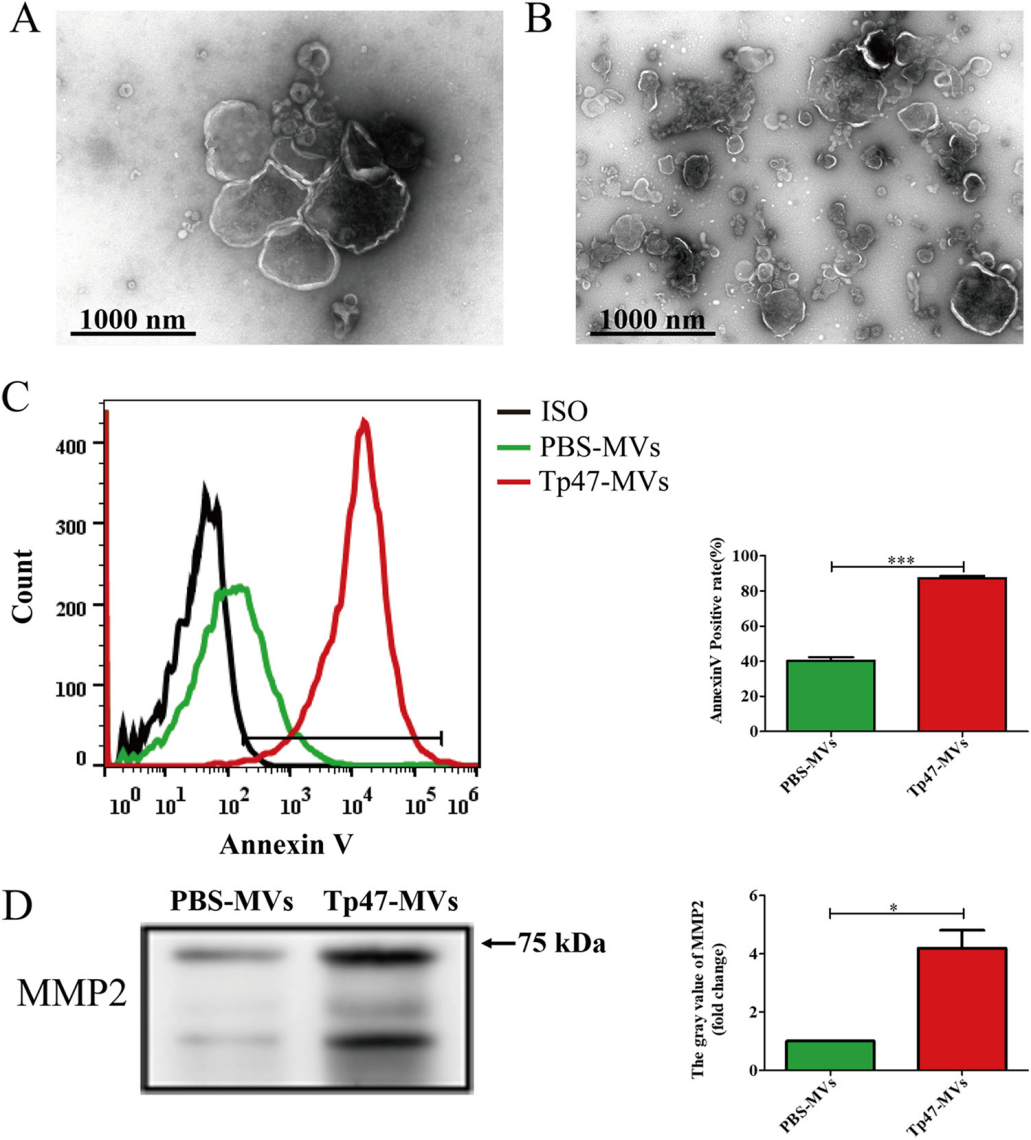
Identification of THP-1 cell-derived microvesicles. (A and B) Morphology of PBS-microvesicles (A) and Tp47-microvesicles (B) shown by transmission electron microscopy. (C) Annexin V-stained microvesicles were detected by flow cytometry. (D) Western blotting was used to determine MMP2 protein expression levels in microvesicles. Student’s *t* test was used to compare intergroup differences. Data were presented as the mean ± SD of three biological replicates. ISO, Isotype control; PBS-MVs, PBS-microvesicles; Tp47-MVs, Tp47-microvesicles; *, *P < *0.05; ***, *P < *0.001.

Microvesicles were isolated from the cell culture medium of THP-1 cells that were stimulated with phosphate-buffered saline (PBS) or 10 μg/mL Tp47 for 24 h. Transmission electron microscopy revealed cup-shaped vesicles, which corroborated the microvesicular morphology (Fig. 1A and 1B). Compared with PBS-stimulated microvesicles (PBS-microvesicles), flow cytometric analysis revealed a significant increase in annexin V positivity in Tp47-microvesicles (*t* = 17.38; *P < *0.001) (Fig. 1C), and Western blotting showed that the protein level of matrix metalloproteinase 2 (MMP2) was high in the vesicles (Fig. 1D).

### Tp47-microvesicles promoted the adhesion of THP-1 cells to HUVECs.

**FIG 2:**
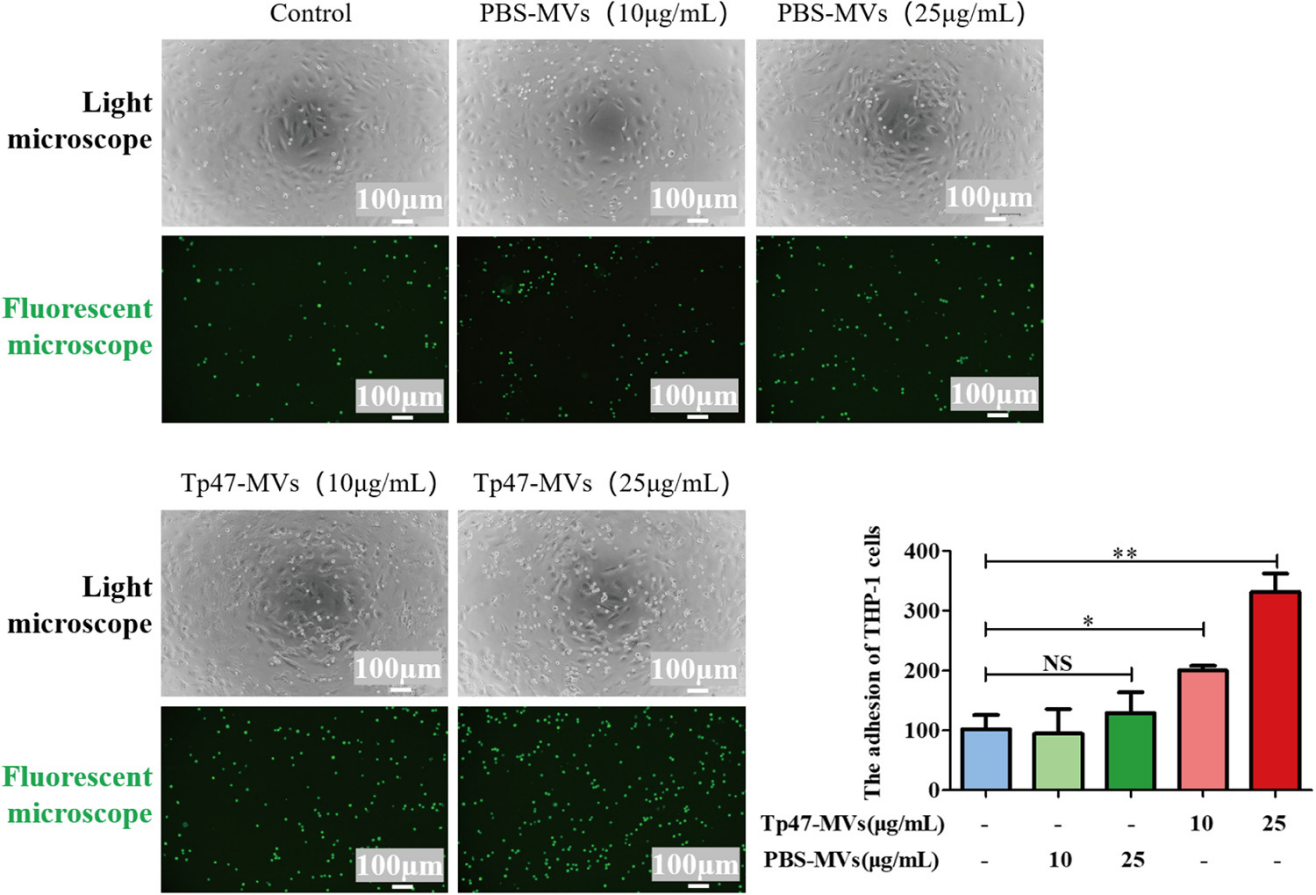
Tp47-microvesicles (Tp47-MVs) promoted the adhesion of THP-1 cells to HUVECs. Images in the top and bottom panels were observed by light and fluorescence microscopy, respectively. The number of THP-1 cells that adhered to HUVECs was determined using ImageJ. Multigroup comparisons were conducted using one-way ANOVA. Data were presented as the mean ± SD of three biological replicates. PBS-MVs, PBS-microvesicles; NS, no significance; *, *P < *0.05; **, *P < *0.01.

To investigate the influence of Tp47-microvesicles on THP-1 cell adherence to HUVECs, HUVECs were pretreated for 24 h with Tp47-microvesicles and PBS-microvesicles at different concentrations and then incubated with THP-1 cells for 1 h. As shown in Fig. 2, Tp47-microvesicles dose-dependently enhanced THP-1 cell adhesion to HUVECs: the number of adherent THP-1 cells was significantly increased in the presence of 10 μg/mL Tp47-microvesicles (*P < *0.05) or 25 μg/mL Tp47-microvesicles (*P < *0.01), which indicated the promotion of the adhesion of THP-1 cells to HUVECs by Tp47-microvesicles.

### Tp47-microvesicles promoted THP-1 cell adhesion to HUVECs via ICAM-1 and VCAM-1.

**FIG 3:**
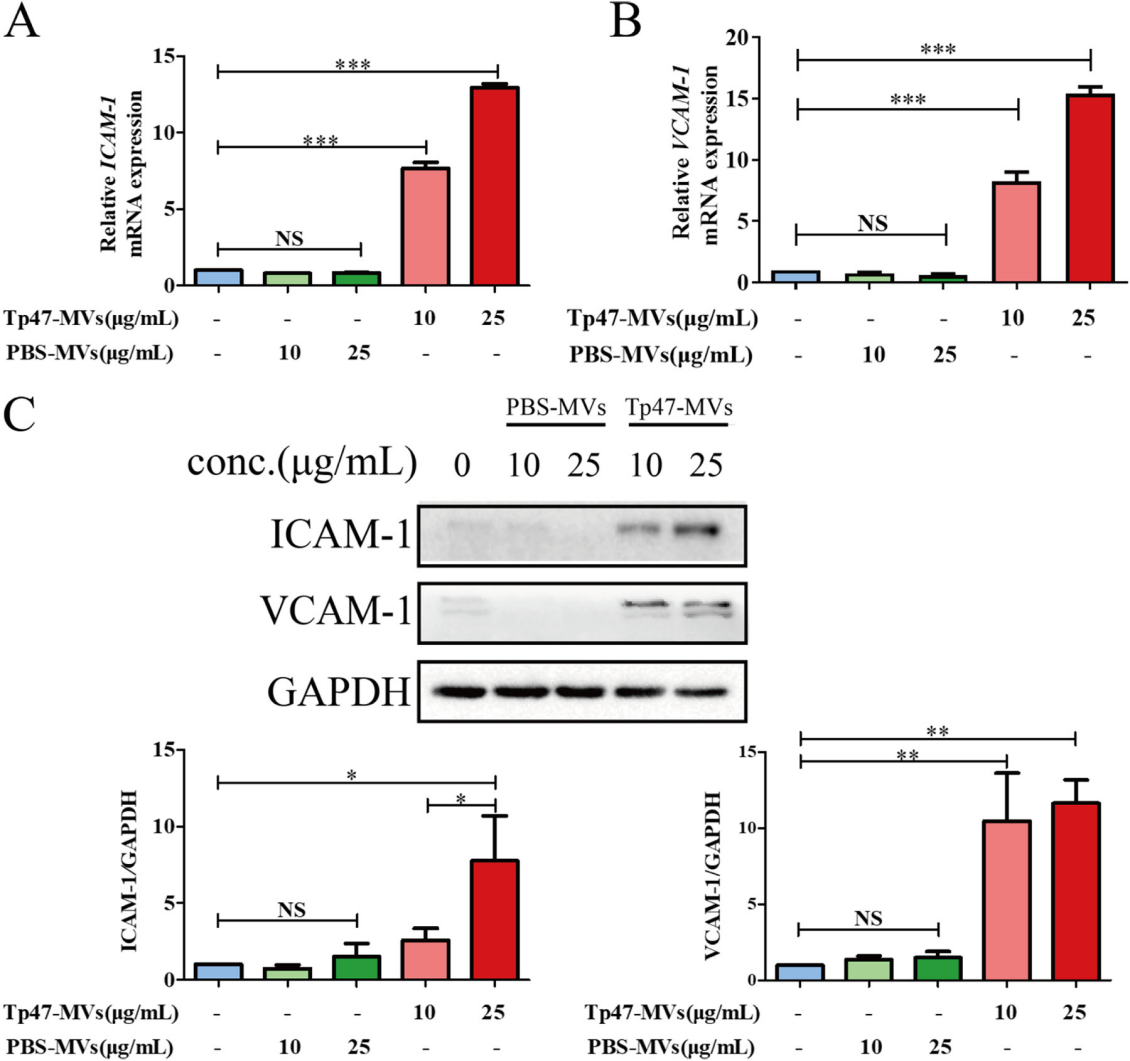
Tp47-microvesicles (Tp47-MVs) induced ICAM-1 and VCAM-1 expression in HUVECs at different doses. (A and B) RT-PCR results for changes in the mRNA expression levels of ICAM-1 and VCAM-1. (C) Western blotting was used to observe the changes in the protein levels of ICAM-1 and VCAM-1. Multigroup comparisons were undertaken using one-way ANOVA. Data were presented as the mean ± SD of three biological replicates. GAPDH, glyceraldehyde-3-phosphate dehydrogenase; PBS-MVs, PBS-microvesicles; NS, no significance; *, *P < *0.05; **, *P < *0.01; ***, *P < *0.001.

To analyze the effects of Tp47-microvesicles on the expression of two cell adhesion-associated genes, *ICAM-1* and *VCAM-1*, HUVECs were pretreated with 10 and 25 μg/mL Tp47-microvesicles or PBS-microvesicles for 24 h. Treatment with Tp47-microvesicles markedly increased the expression of ICAM-1 and VCAM-1 mRNAs in HUVECs (*P < *0.001) (Fig. 3A and B), whereas the protein levels of ICAM-1 and VCAM-1 were dose-dependently increased when HUVECs were stimulated with increasing concentrations of Tp47-microvesicles (Fig. 3C). In contrast, PBS-microvesicles induced no significant change in the mRNA and protein levels of ICAM-1 and VCAM-1 in HUVECs regardless of the dose changes.

**FIG 4:**
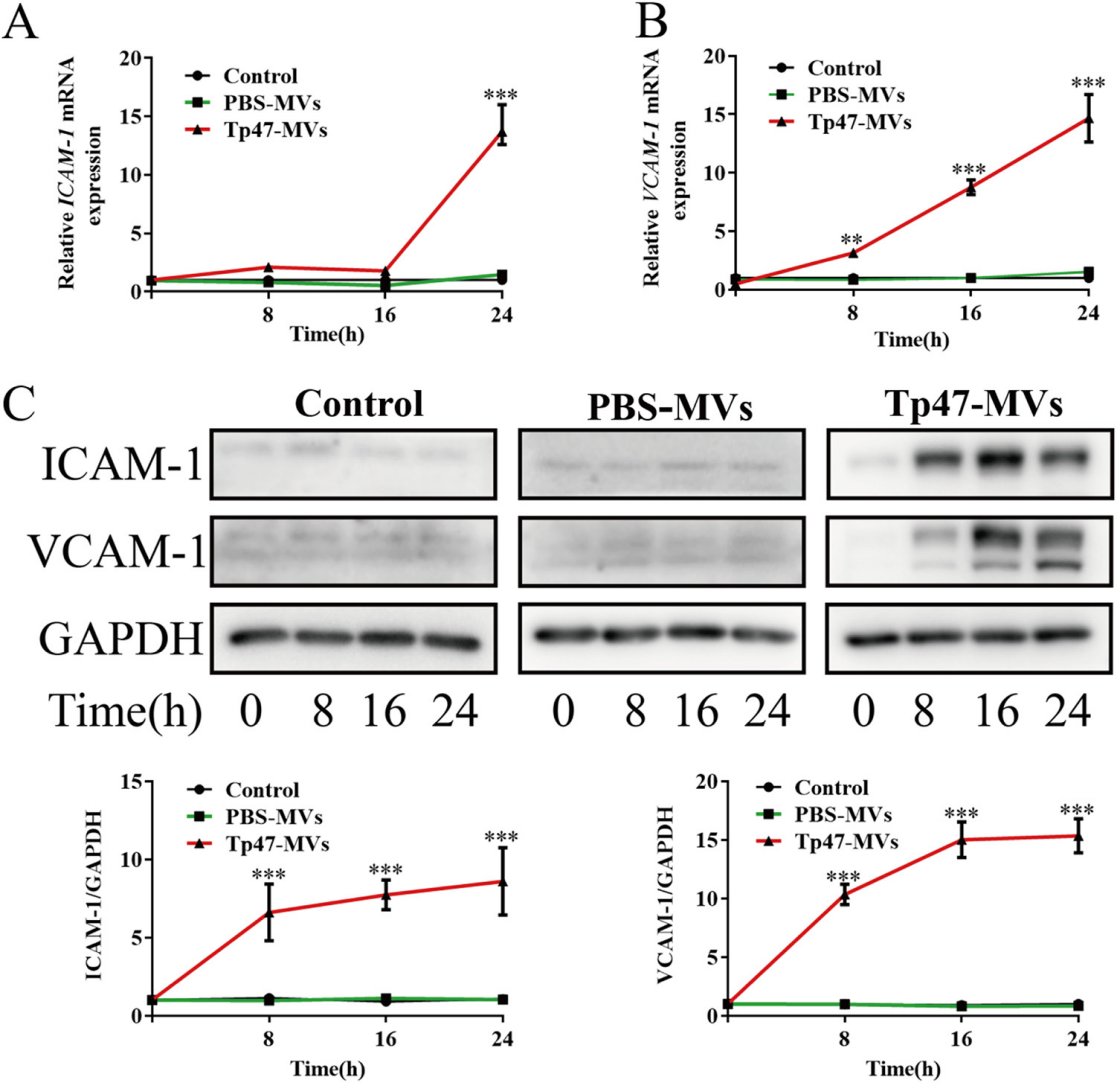
Tp47-microvesicle-induced expression of ICAM-1 and VCAM-1 in HUVECs at different time points. (A and B) Changes in the mRNA expression levels of ICAM-1 and VCAM-1 determined by RT-PCR. (C) Changes in the protein levels of ICAM-1 and VCAM-1 detected by Western blotting. Multigroup comparisons were undertaken using one-way ANOVA. Data were presented as the mean ± SD of three biological replicates. PBS-MVs, PBS-microvesicles; Tp47-MVs, Tp47-microvesicles; **, *P < *0.01; ***, *P < *0.001.

**FIG 5:**
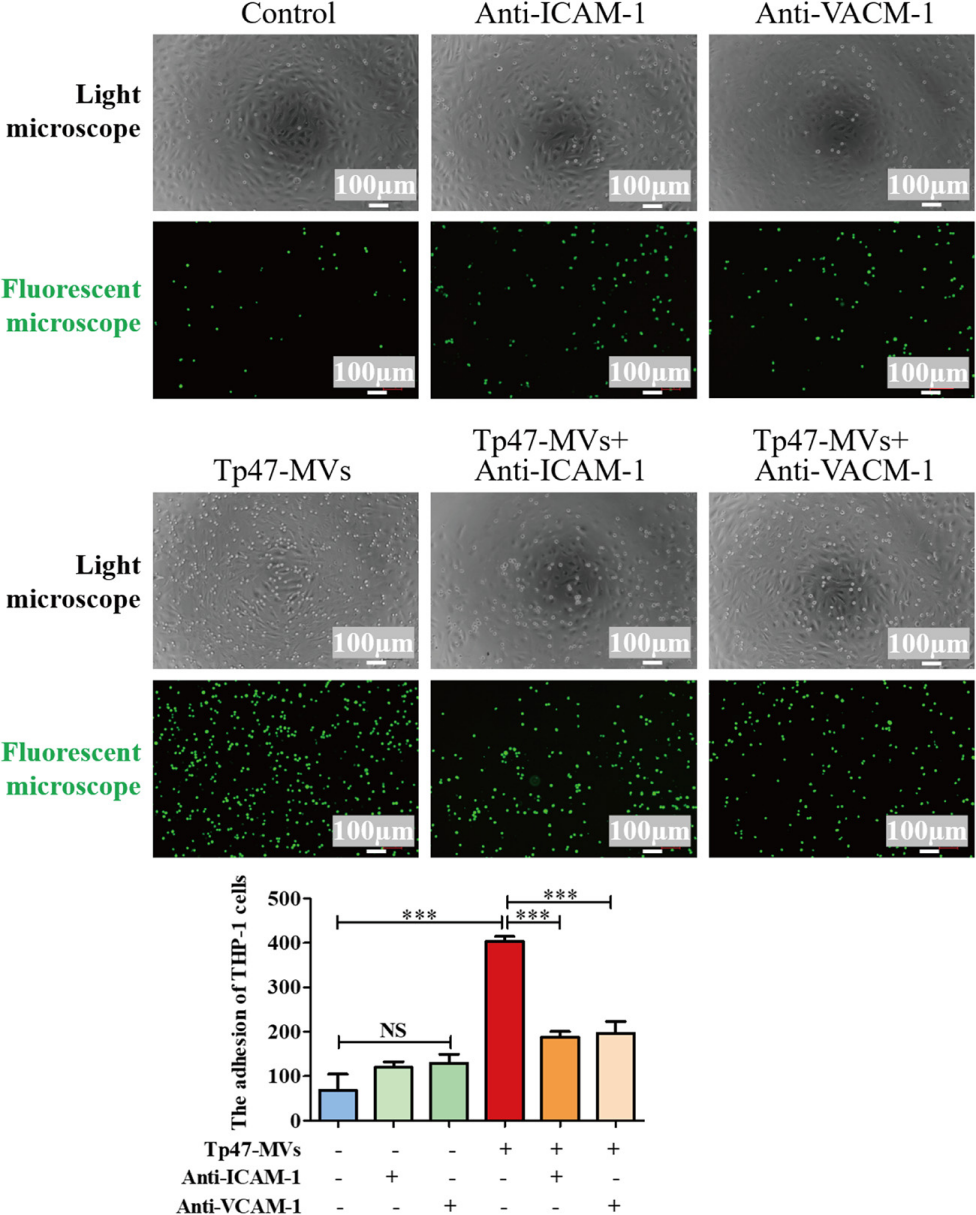
Tp47-microvesicles (Tp47-MVs) promoted the adhesion of THP-1 cells to HUVECs via ICAM-1 and VCAM-1. Optical/fluorescence microscopy revealed the adhesion of THP-1 cells to HUVECs when HUVECs were incubated with 10 μg/mL of an anti-ICAM-1 antibody or an anti-VCAM-1 neutralizing antibody. ImageJ was used to calculate the number of THP-1 cells that adhered to HUVECs. Multigroup comparisons were undertaken using one-way ANOVA. Data were presented as the mean ± SD of three biological replicates. **, *P < *0.01; ***, *P < *0.001.

Additionally, HUVECs were incubated with 25 μg/mL PBS-microvesicles or Tp47-microvesicles for 0, 8, 16, and 24 h. In the Tp47-microvesicle group, the ICAM-1 mRNA levels were increased significantly at 24 h (*P < *0.001), whereas the VCAM-1 mRNA levels were increased considerably at 8, 16, and 24 h (*P < *0.01 to 0.001) (Fig. 4A and B). Furthermore, we observed significant increases in ICAM-1 and VCAM-1 protein expression at 8, 16, and 24 h (*P < *0.001) (Fig. 4C). In contrast, compared with the control group, PBS-microvesicles did not significantly alter the expression of adhesion molecules regardless of the incubation time (Fig. 4A to C). To identify the cell surface molecules that mediate THP-1 cell adhesion to HUVECs, an anti-ICAM-1 antibody and an anti-VCAM-1 neutralizing antibody were used to block ICAM-1 and VCAM-1, respectively. Thereafter, the adherence of THP-1 cells to HUVECs was suppressed significantly, and this indicated that Tp47-microvesicles promote THP-1 cell adhesion to HUVECs via ICAM-1 and VCAM-1 (Fig. 5).

### Tp47-microvesicles activated the ERK1/2 and NF-κB pathways in HUVECs.

**FIG 6:**
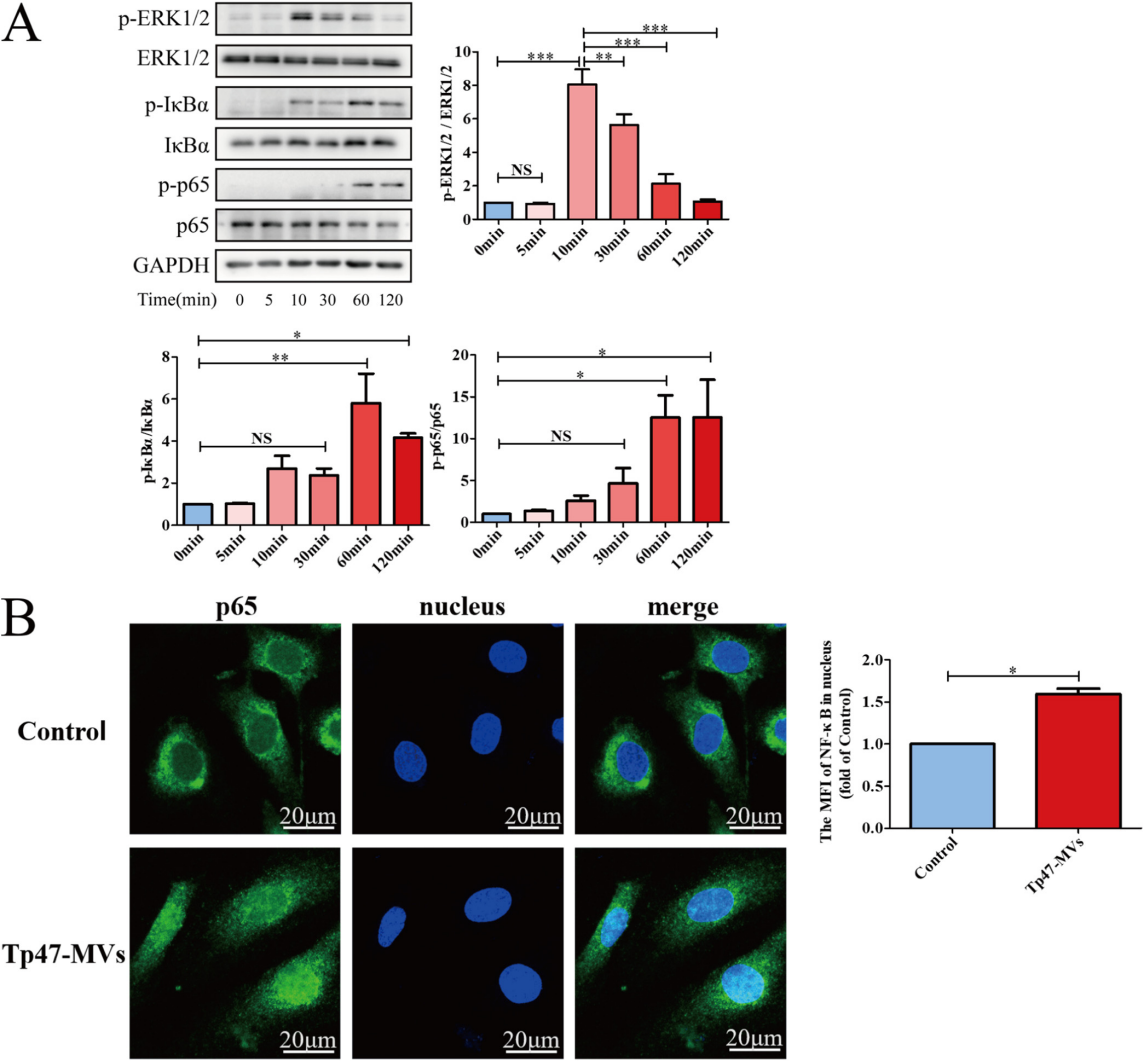
Tp47-microvesicles (Tp47-MVs) activated the ERK1/2 and NF-κB signaling pathways in HUVECs. (A) Phosphorylated and total protein levels of ERK1/2, IκBα, and p65. (B) Nuclear translocation of the NF-κB p65 subunit. Multigroup comparisons were undertaken using one-way ANOVA. Data were presented as the mean ± SD of three biological replicates. MFI, mean fluorescence intensity; NS, no significance; *, *P < *0.05; **, *P < *0.01; ***, *P < *0.001.

To investigate the mechanism by which Tp47-microvesicles induce the adherence of THP-1 cells to HUVECs, the extracellular signal-regulated kinase 1/2 (ERK1/2) and NF-κB proteins were first evaluated by Western blotting. When HUVECs were incubated with Tp47-microvesicles, ERK1/2 phosphorylation increased at 10 min (*P < *0.05) and subsequently decreased over time; IκBα and p65 phosphorylation increased at 60 min (*P < *0.01) and 120 min (*P < *0.05) (Fig. 6A). Furthermore, an immunofluorescence-based method revealed that Tp47-microvesicles induced the translocation of the NF-κB p65 subunit into the HUVEC nuclei (Fig. 6B).

**FIG 7:**
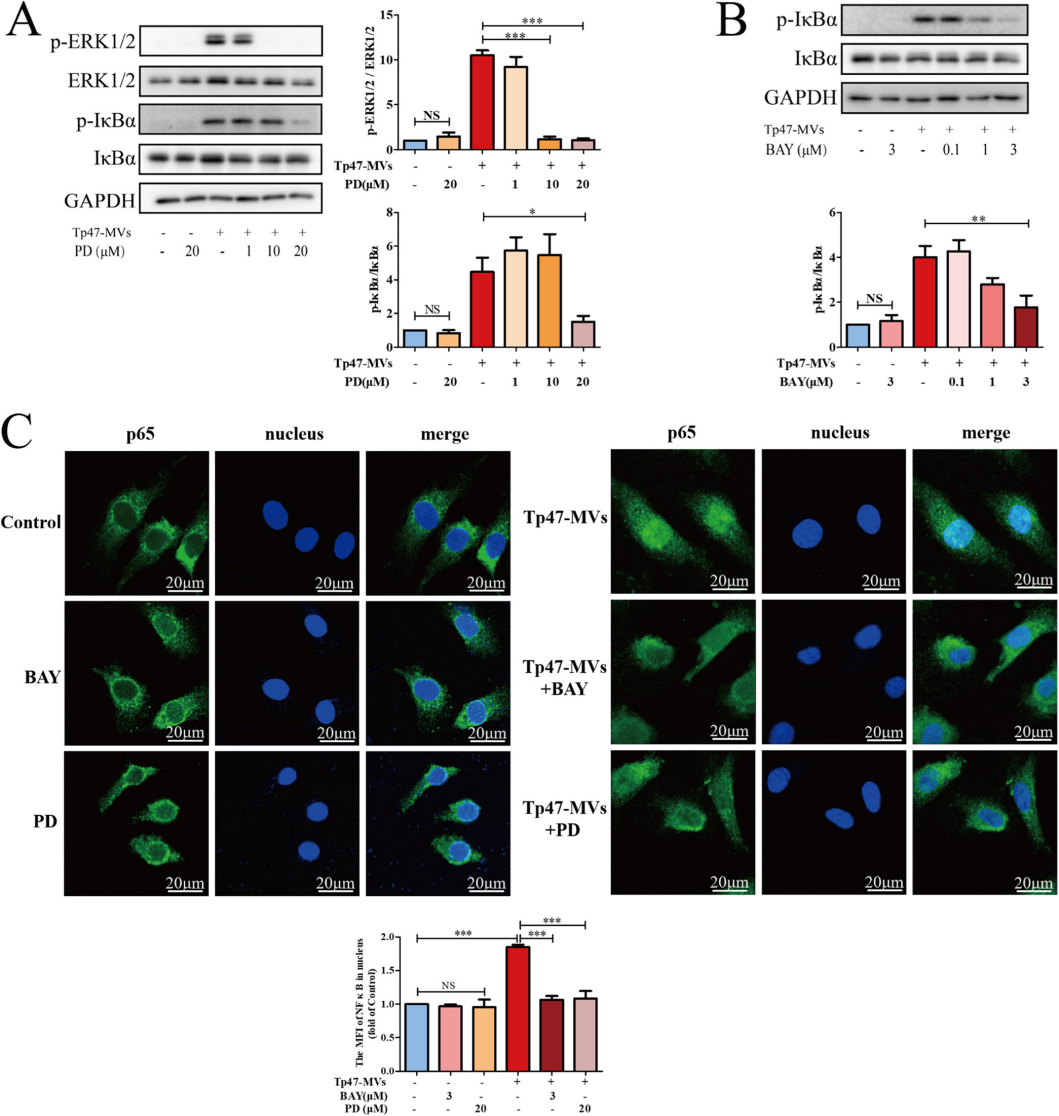
NF-κB transcriptional activity was mediated by the ERK1/2 signaling pathway. (A) HUVECs were preincubated with PD98059 (PD), and the phosphorylation of ERK1/2 and IκBα was ascertained by Western blotting. (B) After the preincubation of HUVECs with BAY11-07085 (BAY), IκBα phosphorylation was observed by Western blotting. (C) Nuclear translocation of the NF-κB p65 subunit. Multigroup comparisons were undertaken using one-way ANOVA. Data were presented as the mean ± SD of three biological replicates. Tp47-MVs, Tp47-microvesicles; MFI, mean fluorescence intensity; NS, no significance; *, *P < *0.05; **, *P < *0.01; ***, *P < *0.001.

To determine the association between ERK1/2 and NF-κB, the ERK1/2 and NF-κB pathway inhibitors PD98059 and BAY11-7085, respectively, were incubated with HUVECs for 1 h. The phosphorylation of ERK1/2 was measured following stimulation of HUVECs with Tp47-microvesicles for 10 min and the phosphorylation of IκBα was measured following stimulation of HUVECs with Tp47-microvesicles for 1 h. Preincubation with PD98059 significantly suppressed the Tp47-microvesicle-induced phosphorylation of ERK1/2 and IκBα, whereas preincubation with BAY11-7085 significantly suppressed IκBα phosphorylation (Fig. 7A and B). As illustrated in Fig. 7C, pretreatment with PD98059 or BAY11-7085 inhibited the Tp47-microvesicle-induced nuclear translocation of the NF-κB p65 subunit in HUVECs.

### Components of the ERK1/2 and NF-κB signaling pathways are essential for Tp47-microvesicle-induced adhesion of THP-1 cells to HUVECs.

**FIG 8:**
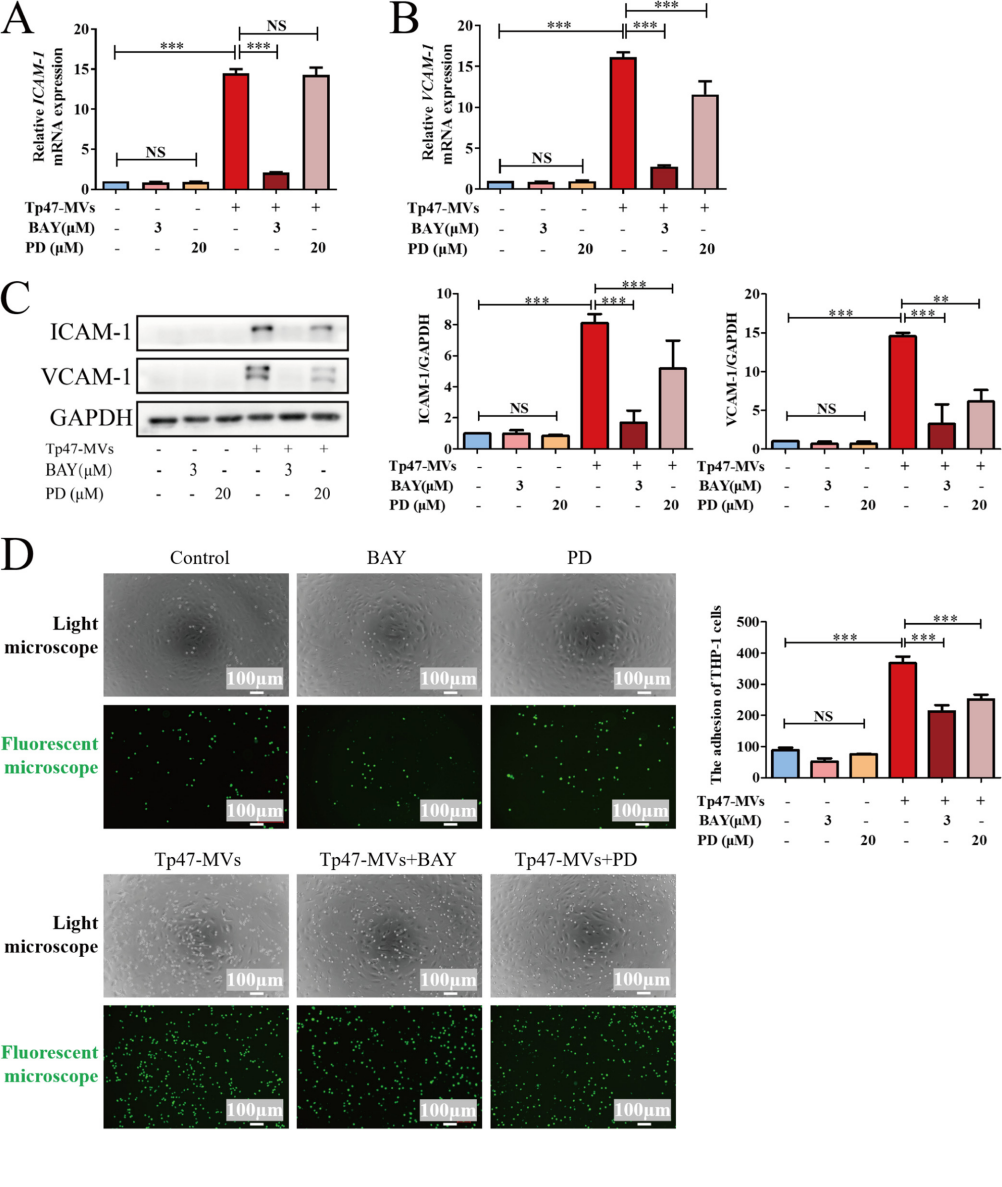
Components of the ERK1/2 and NF-κB signaling pathways are essential for Tp47-microvesicle-induced adhesion of THP-1 cells to HUVECs. (A and B) Changes in the mRNA expression levels of ICAM-1 and VCAM-1 determined by RT-PCR. (C) Changes in the ICAM-1 and VCAM-1 protein levels detected by Western blotting. (D) Preincubation of HUVECs with PD98059 (PD) and BAY11-7085 (BAY) inhibited the adhesion of THP-1 cells to HUVECs. Optical/fluorescence microscopy revealed the adherence of THP-1 cells to HUVECs. Multigroup comparisons were undertaken using one-way ANOVA. Data were presented as the mean ± SD of three biological replicates. Tp47-MVs, Tp47-microvesicles; NS, no significance; *, *P < *0.05; **, *P < *0.01; ***, *P < *0.001.

Notably, the NF-κB inhibitor BAY11-07085 prevented the Tp47-microvesicle-induced mRNA expression of ICAM-1 and VCAM-1. The ERK1/2 inhibitor PD98059 attenuated VCAM-1 but not ICAM-1 mRNA expression (Fig. 8A and B). Pretreatment with PD98059 and BAY11-7085 attenuated Tp47-microvesicle-induced ICAM-1 and VCAM-1 protein expression (Fig. 8C). The pretreatment of HUVECs with PD98059 and BAY11-07085 attenuated the Tp47-microvesicle-induced adhesion of THP-1 cells to HUVECs (Fig. 8D). These results indicated that the ERK1/2 and NF-κB pathways are essential for the Tp47-microvesicle-induced adhesion of THP-1 cells to HUVECs.

## DISCUSSION

The present consensus among syphilologists is that the clinical manifestations of syphilitic infection emerge from treponeme-driven inflammatory processes (16). Furthermore, the pathology of syphilis is characterized by vascular manifestations such as periarteritis and endarteritis. As the T. pallidum genome does not encode orthologs of either well-characterized bacterial toxins or components of secretory systems (17), membrane proteins are considered the virulence factors of T. pallidum. Tp47 induces the adherence of THP-1 cells to human dermal vascular smooth muscle cells by increasing adhesion-associated cytokine production (10). Microvesicles belong to a group of heterogeneous membrane-coated vesicles that can function as signaling components in inflammatory processes (18). However, it is unclear whether microvesicles are involved and mediate the interaction of immunocytes with vascular cells to contribute to vascular inflammation. This study showed that Tp47-microvesicles promoted THP-1 cell adherence to HUVECs and increased the mRNA and protein expression levels of ICAM-1 and VCAM-1. Moreover, the adherence of THP-1 cells to HUVECs was inhibited considerably by anti-ICAM-1 and anti-VCAM-1 neutralizing antibodies. Furthermore, Tp47-microvesicle treatment of HUVECs activated the ERK1/2 and NF-κB signaling pathways, which induced ICAM-1 and VCAM-1 expression. These findings confirmed that Tp47-microvesicles increase the adhesion of THP-1 cells to HUVECs through the induction of ICAM-1 and VCAM-1 expression via the activation of the ERK1/2 and NF-κB pathways.

Endothelial dysfunction, an early phase of vascular inflammation in humans, commences with ICAM-1 and VCAM-1 expression and immune cell adhesion (5, 19, 20). In this study, we found that Tp47-microvesicles enhanced THP-1 cell adherence to HUVECs and upregulated ICAM-1 and VCAM-1 mRNA and protein expression, which may contribute to syphilitic vascular inflammation. The identification of the mechanisms that underlie the interaction of immune cells with HUVECs that may downregulate abnormal leukocytic adhesion in the vascular endothelium may serve to prevent and treat T. pallidum-induced vascular inflammation.

Recently, evidence of the importance of extracellular vesicles for intercellular communication processes, with key implications for endothelial homeostasis, cell survival, inflammation, and thrombosis, has been reported (21). The cell adhesion molecules ICAM-1 and VCAM-1 are expressed in HUVECs by microvesicles in lipopolysaccharide-treated THP-1 cells (22). In this study, we found that the coincubation of Tp47-microvesicles with HUVECs increased THP-1 cell adhesion to HUVECs and upregulated ICAM-1 and VCAM-1 expression. Moreover, the increased adhesion of THP-1 cells to HUVECs was inhibited by anti-ICAM-1 and anti-VCAM-1 neutralizing antibodies, suggesting that the increased expression of ICAM-1 and VCAM-1 increased THP-1 cell adhesion. Thus, microvesicles participate in the Tp47-induced recruitment of inflammatory cells in the immunopathogenesis of syphilis, and the Tp47-microvesicle-induced expression of ICAM-1 and VCAM-1 in HUVECs may be a crucial factor in the T. pallidum-induced vascular inflammatory response through the recruitment of monocytes to inflammatory areas.

Cell adhesion is regulated by a network that comprises cell signaling molecules, epigenetic mechanisms, transcription factors, and posttranscriptional regulators. ICAM-1 expression is elicited by the activation of a redox-sensitive intracellular signaling cascade that involves ERK1/2 and p38 mitogen-activated protein kinase (MAPK) and results in NF-κB activation (23). In this study, the ERK1/2 inhibitor PD98059 inhibited the protein expression of adhesion molecules and impaired THP-1 cell adhesion to HUVECs. The pretreatment of HUVECs with the ERK1/2 inhibitor PD98059 inhibited the Tp47-microvesicle-induced phosphorylation of ERK1/2 and the nuclear translocation of NF-κB. Although the ERK1/2 inhibitor PD98059 failed to inhibit ICAM-1 mRNA expression, possibly through translational rather than transcriptional effects, these findings indicated that Tp47-microvesicles mediate THP-1 cell adhesion to HUVECs in an ERK1/2-dependent manner.

The activation of the transcription factor NF-κB is primarily responsible for the inflammatory response that occurs after exposure to stimuli. IκBα sequesters NF-κB in the cytoplasm, and IκBα phosphorylation induces the proteasome-mediated degradation of IκBα and the subsequent activation and nuclear translocation of NF-κB; this mechanism is a key activator of the genes that encode cytokines and adhesion molecules (23). In this study, Tp47-induced ICAM-1 and VCAM-1 expression and THP-1 cell adhesion were completely abolished by the NF-κB pathway inhibitor BAY11-7085. This indicates that Tp47-microvesicle-stimulated IκBα phosphorylation and NF-κB nuclear translocation are crucial factors for ICAM-1 and VCAM-1 upregulation and mediate the adherence of THP-1 cells to HUVECs. In addition, the ERK1/2 inhibitor PD98059 hindered IκBα phosphorylation, whereas BAY11-7085 did not block ERK1/2 phosphorylation (data not shown). These findings suggest that the activation of an intracellular signaling cascade involving ERK1/2, culminating in the activation of NF-κB signals, is the cause of TP47-microvesicle-induced ICAM-1 and VCAM-1 expression.

Several potential limitations of this study should be acknowledged. First, based on centrifugation protocols, the separation of exosomes usually requires a centrifugal force of more than 100,000 × *g* (24); however, the microvesicles were collected at only 20,000 × *g*, and thus, it is inevitable that microvesicles with exosomes were isolated. Second, extracellular vesicles represent an important mode of intercellular communication as vehicles for the cell-cell transfer of membrane and cytosolic proteins, lipids, and RNA (25). Proteomic analysis of microvesicles is needed to determine their biophysical and biochemical properties. Finally, there is the limitation that microvesicles secreted by individual immune cells cannot mimic the infection process in a syphilitic host. Therefore, animal experiments are preferable for comprehensively ascertaining the role of microvesicles in vascular inflammation and the pathogenesis of syphilis.

### Conclusion.

**FIG 9:**
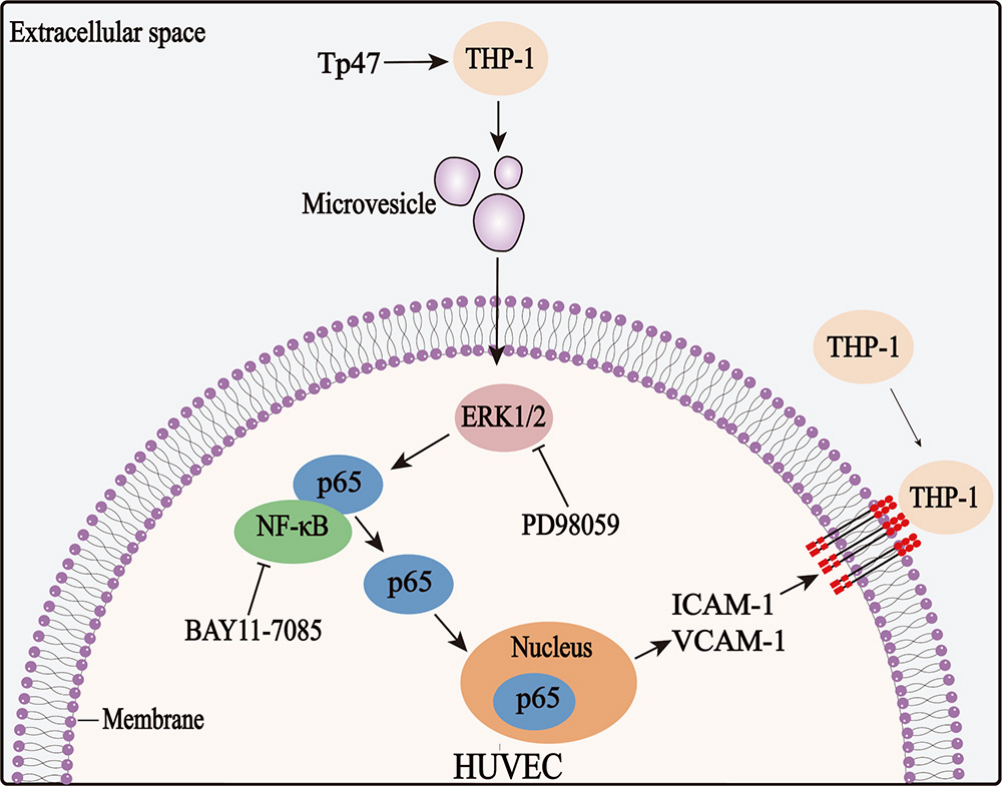
Schematic diagram of the signaling pathways involved in the Tp47-microvesicle-induced adhesion of THP-1 cells to HUVECs via the upregulation of ICAM-1 and VCAM-1 expression.

In summary, this study showed that Tp47-microvesicles promote the adherence of THP-1 cells to HUVECs by inducing ICAM-1 and VCAM-1 expression. This suggests that microvesicles constitute a functional inflammatory mediator between vascular cells and immunocytes and thereby contribute to vascular inflammation in syphilis. Furthermore, the above-mentioned process is mediated by the ERK1/2 and NF-κB signaling pathways (Fig. 9). Recognition of the relevant mechanisms of ICAM-1 and VCAM-1 expression and monocyte adhesion may enable a better understanding of the pathophysiology of syphilitic vascular inflammation.

## MATERIALS AND METHODS

### Preparation of the recombinant T. pallidum Tp47 protein.

The endotoxin in the recombinant T. pallidum Tp47 protein (Boson Biotech Co., Ltd., Xiamen, China) was removed as described previously (8). Endotoxin contamination of the Tp47 preparation was quantified using *Tachypleus* amebocyte lysate (Chinese Horseshoe Crab Reagent Manufactory, Ltd., Xiamen, China), which detected <0.05 endotoxin units (EU)/mL.

### Cell culture.

HUVECs (ScienCell Research Laboratories, Carlsbad, CA, USA) were incubated in endothelial cell medium, which contained 5% fetal bovine serum (FBS) and 1% endothelial cell growth supplement (ScienCell Research Laboratories, Carlsbad, CA, USA). THP-1 cells (American Type Culture Collection, Manassas, VA, USA) were cultured in RPMI 1640 medium (American Type Culture Collection), which was supplemented with 10% fetal bovine serum (American Type Culture Collection), 1% penicillin, and streptomycin at 37°C with 5% CO_2_. Next, the cell culture dishes were transferred to serum-free medium, and THP-1 cells were stimulated with PBS or 10 μg/mL Tp47 for 24 h; thereafter, culture media were collected for microvesicle isolation.

### Isolation of microvesicles.

THP-1 cells (1 × 10^6^ cells/mL) were processed with PBS or 10 μg/mL Tp47 for 24 h in culture medium as described above. The cells were centrifuged at 500 × *g* for 10 min at 4°C. The supernatant was collected and centrifuged at 2,000 × *g* for 20 min to ensure the pelleting of the cellular debris. The microvesicle pellets were obtained by centrifuging the microvesicle-containing supernatant at 20,000 × *g* for 30 min at 4°C (24). The microvesicle pellets were washed, resuspended in PBS, and stored at −80°C until use. Microvesicles derived from Tp47-stimulated or PBS-stimulated THP-1 cells were denoted Tp47-microvesicles or PBS-microvesicles, respectively. The microvesicle concentration was quantified by measuring the total protein concentration using a bicinchoninic acid (BCA) protein assay kit (TaKaRa, Shanghai, China).

### Identification of microvesicles.

To observe the morphology of the microvesicles, the samples were examined by transmission electron microscopy using a negative staining method as previously described (26). Microvesicles were stained with annexin V (a marker of microvesicles [27]) and detected by flow cytometry. Briefly, microvesicles were stained with annexin V-fluorescein isothiocyanate (FITC) (Beyotime, Shanghai, China) in annexin V-FITC binding buffer for 30 min in the dark at room temperature. The samples were then centrifuged at 20,000 × *g* for 15 min at 4°C. After the removal of the supernatant, the microvesicles were resuspended in 200 μL PBS and immediately subjected to fluorescence-activated cell sorting (FACS), wherein the percentage of annexin-positive microvesicles was analyzed by utilizing a FACSCanto II flow cytometer (BD Biosciences, San Diego, CA, USA). The results were analyzed using FlowJo7.6.4 software (TreeStar, Ashland, OR, USA). Western blot analysis was used to quantify the concentration of matrix metalloproteinase 2 (MMP2) (another marker of microvesicles [28]) in microvesicles as described previously (29).

### Analysis of ICAM-1 and VCAM-1 expression in Tp47-microvesicle-treated HUVECs.

HUVECs were stimulated with different doses (0, 10, and 25 μg/mL for 24 h) of Tp47-microvesicles and PBS-microvesicles or for different durations (0, 8, 16, and 24 h with 25 μg/mL Tp47-microvesicles and PBS-microvesicles). The posttreatment mRNA levels of ICAM-1 and VCAM-1 were measured by reverse transcription-PCR (RT-PCR) using previously described primers for real-time PCR analysis. The relative mRNA expression was presented as normalised VCAM-1 and ICAM-1 mRNA to glyceraldehyde 3-phosphate dehydrogenase (GAPDH) mRNA by the 2^–ΔΔCT^method (10, 30). The levels of the ICAM-1 and VCAM-1 proteins in HUVECs were determined by Western blotting.

### Adherence assay.

HUVECs were treated with different doses of Tp47-microvesicles and PBS-microvesicles (0, 10, and 25 μg/mL) for 24 h at 37°C with 5% CO_2_. After prestaining with 10 μM calcein AM for 30 min at 37°C, THP-1 cells were added to HUVEC culture plates and incubated at 37°C for 1 h. After three washes with RPMI 1640 medium, the adherent THP-1 cells were observed by fluorescence microscopy and counted using ImageJ software. For the inhibition experiments, HUVECs were preincubated with anti-ICAM-1 or anti-VCAM-1 neutralizing antibody (10 μg/mL; R&D Systems, Inc., Minneapolis, MN, USA), the NF-κB inhibitor BAY11-7085 (3 μM; MedChemExpress, NJ, USA), or the ERK1/2 inhibitor PD98059 (20 μM; MedChemExpress) for 1 h, and the adherence assay was performed as described above.

### Analysis of ERK1/2 and NF-κB activation in Tp47-microvesicle-treated HUVECs.

HUVECs were stimulated with 25 μg/mL Tp47-microvesicles for various durations, and the cell lysates were collected for Western blotting to detect phosphorylated and total ERK1/2, IκBα, and p65 proteins. To confirm the Tp47-microvesicle-induced activation of the ERK1/2 and NF-κB signaling pathways, HUVECs were preincubated with the ERK1/2 inhibitor PD98059 and the NF-κB inhibitor BAY11-7085 for 1 h before stimulation with 25 μg/mL Tp47-microvesicles. The expression of phosphorylated and total proteins of the ERK1/2 and NF-κB signaling pathways was detected. For determining the ICAM-1 and VCAM-1 expression levels, the cells were stimulated for 24 h as described above. The anti-phosphorylated ERK1/2 (p-ERK1/2), anti-p-IκBα, anti-p-p65, anti-ERK1/2, anti-IκBα, and anti-p65 antibodies were obtained from Cell Signaling Technology (Danvers, MA, USA).

### Analysis of the nuclear translocation of the NF-κB p65 subunit.

HUVECs cultured on a Millicell EZ Slide 4-well glass slide box (Millipore, Burlington, MA, USA) were stimulated with 25 μg/mL Tp47-microvesicles for 1 h, and the nuclear translocation of the NF-κB p65 subunit was analyzed as mentioned previously (2). For the inhibition assay, HUVECs pretreated with 3 μM BAY11-7085 and 20 μM PD98059 for 1 h were stimulated with 25 μg/mL Tp47-microvesicles for 1 h, and the nuclear translocation of the NF-κB p65 subunit was analyzed.

### Statistical analysis.

All data in this research are presented as means ± standard deviations (SD). Student’s *t* test or one-way analysis of variance (ANOVA) was used to compare two or multiple groups, respectively. All statistical analyses were performed using GraphPad Prism 8.0 (GraphPad Software, La Jolla, CA, USA). *P* values of <0.05 were deemed statistically significant.

### Data availability.

The data that support the findings of this study are available from the corresponding authors upon reasonable request.
